# Automatic contour segmentation of cervical cancer using artificial intelligence

**DOI:** 10.1093/jrr/rrab070

**Published:** 2021-08-14

**Authors:** Yosuke Kano, Hitoshi Ikushima, Motoharu Sasaki, Akihiro Haga

**Affiliations:** Department of Radiological Technology, Tokushima Prefecture Naruto Hospital, 32 Kotani, Muyacho, Kurosaki, Naruto-shi, Tokushima 772-8503, Japan; Department of Therapeutic Radiology, Institute of Biomedical Sciences, Tokushima University Graduate School, 3-18-15 Kuramoto-Cho, Tokushima, Tokushima 770-8503, Japan; Department of Therapeutic Radiology, Institute of Biomedical Sciences, Tokushima University Graduate School, 3-18-15 Kuramoto-Cho, Tokushima, Tokushima 770-8503, Japan; Department of Medical Image Informatics, Institute of Biomedical Sciences, Tokushima University Graduate School, 3-18-15 Kuramoto-Cho, Tokushima, Tokushima 770-8503, Japan

**Keywords:** cervical cancer, automatic tumor contour segmentation, radiation therapy, diffusion-weighted imaging (DWI), Dice similarity coefficient (DSC)

## Abstract

In cervical cancer treatment, radiation therapy is selected based on the degree of tumor progression, and radiation oncologists are required to delineate tumor contours. To reduce the burden on radiation oncologists, an automatic segmentation of the tumor contours would prove useful. To the best of our knowledge, automatic tumor contour segmentation has rarely been applied to cervical cancer treatment. In this study, diffusion-weighted images (DWI) of 98 patients with cervical cancer were acquired. We trained an automatic tumor contour segmentation model using 2D U-Net and 3D U-Net to investigate the possibility of applying such a model to clinical practice. A total of 98 cases were employed for the training, and they were then predicted by swapping the training and test images. To predict tumor contours, six prediction images were obtained after six training sessions for one case. The six images were then summed and binarized to output a final image through automatic contour segmentation. For the evaluation, the Dice similarity coefficient (DSC) and Hausdorff distance (HD) was applied to analyze the difference between tumor contour delineation by radiation oncologists and the output image. The DSC ranged from 0.13 to 0.93 (median 0.83, mean 0.77). The cases with DSC <0.65 included tumors with a maximum diameter < 40 mm and heterogeneous intracavitary concentration due to necrosis. The HD ranged from 2.7 to 9.6 mm (median 4.7 mm). Thus, the study confirmed that the tumor contours of cervical cancer can be automatically segmented with high accuracy.

## INTRODUCTION

In recent years, the development of artificial intelligence (AI) has made it possible to utilize various technologies such as image recognition, speech recognition, natural language processing and predictive processing. AI has made significant advancements in the medical field, where optimal medical care must be provided sustainably and efficiently. AI has the potential to be utilized in diagnostic imaging support, treatment support, drug development, nursing care, dementia support and surgical support. In the field of radiotherapy, AI can be used for automatic contour segmentation, treatment planning and selection of individual treatments for patients based on treatment outcome predictions.

Automatic contour segmentation is a technique wherein an object of interest is imaged by separating it from the background [1]. This technique is currently used not only in general applications, such as in extracting images of people from backgrounds and separating landscapes, but also in medical applications. In the research stage, clinical applications of this method have been reported for various cases, such as classification of the inside of glioblastoma in the brain using deep learning, classification of lung lobes in normal lungs, recognition of upper and lower limb bones and extraction of gas in the intestinal tract [2–6].

Cervical cancer can be treated by surgery, chemotherapy, radiotherapy or a combination of the two, depending on the degree of tumor progression [7]. When radiation therapy is the treatment of choice, computed tomography (CT) and magnetic resonance imaging (MRI) are used to perform three-dimensional conformal radiotherapy (3D-CRT) planning. The 3D-CRT planning requires on average approximately one hour of work, with the contouring of the tumor and organs at risk (OARs) being essential aspects [8, 9]. In clinical practice, radiation oncologists perform contouring during treatment planning, in addition to other tasks such as medical examinations and reviewing receipts. There is an inter-observer variation in the contour delineation during treatment planning; an inter-observer variation in the contour delineation influence can be reduced using automatic contour segmentation. In recent years, the burden of planning work has increased given the high number of cancer patients associated with aging and the complexity of planning work associated with high-precision technologies employed for irradiation. AI technology can be applied to reduce inter-observer variation while contouring—which is a time consuming and laborious part of radiotherapy planning—and help improve medical practice in this area.

Automatic contour segmentation for prophylactic clinical target volume (CTV) and OARs has been clinically applied using atlas-based and model-based segmentation methods, which are part of commercial radiotherapy planning devices [10, 11]. However, it is difficult to extract the gross tumor volume (GTV) that is ubiquitous in the human body using these segmentation methods, with no clinical applications reported thus far. On the other hand, deep learning has been applied to various cases. For example, in gynecology, Wang *et al.* [12] reported a study on automatic contour segmentation using CT images of the CTV, including prophylactic lymph nodes in cervical cancer. However, to the best of our knowledge, there is no clinical application involving AI-based automatic contour segmentation for the GTV of cervical cancer. The accuracy of GTV contour segmentation in radiotherapy is another essential treatment planning factor because it directly affects the treatment outcome. The automatic contour segmentation of cervical cancer GTVs using deep learning would require GTV contour data for training. Such contour data, which are essential for training, are known to have individual differences depending on the personnel performing the contour delineation [13]. If the contours of the GTV of cervical cancer can be obtained with high accuracy and precision using automated contour segmentation, the artificial influence of the contours of the GTV in the institution can be eliminated. Eliminating the contoured human influence will improve clinical outcomes by reducing the variability in tumor control rates and OAR side effects among patients. Moreover, if automated contour segmentation can be used within and across institutions, it will provide helpful information for treatment facilities in Japan and help to equalize treatment facilities for cervical cancer. Furthermore, application of the findings of this research to other parts of the body, as a pilot study, can be an excellent AI research achievement.

MRI can show the boundaries of cancer more clearly than CT because of the former’s higher intensity resolution. Groupe Européen de Curiethérapie (European Society for Therapeutic Radiology and Oncology) recommends MRI for contouring of the primary tumor volume of cervical cancer in the radiation treatment planning [14]. We focus on diffusion-weighted imaging (DWI) as the first step of our study of auto-contouring since DWI shows the most amount of contrast between tumor and normal tissue in any MRI sequence. Therefore we applied DWI, which is a form of MRI, with high contrast between the GTV and the surrounding OARs to reduce the individual differences in the contours. We collected DWIs of cervical cancer patients undergoing radiotherapy and performed automatic contour segmentation of the GTV using U-Net, a type of deep learning [15, 16].

The learning model used in this study is a combined model of 3D U-Net and 2D U-Net. The 3D U-Net has only one learning model for each case since the number of cases in this study is 98. Therefore, the 3D U-Net model alone cannot be used with the learning model data from many cases. Therefore, we combine 3D U-Net with 2D U-Net to increase the number of target slices for the learning model data. However, in the case of 2D U-Net, the data on continuity due to tumor are lost, which is another reason to combine 3D U-Net with 2D U-Net. Moreover, by using k-fold cross-validation for each model, we can create unbiased teacher data. Furthermore, by using bagging, which is one of the ensemble learning methods, we can expect to output the final region with high confidence from multiple predictions. To the best of our knowledge, no extant study has attempted automatic contour extraction for the GTV of cervical cancer using a combination of 3D U-Net and 2D U-Net. In this study, we compared the results obtained by bagging with the proposed multiple k-fold verification technique with those of automatic contour segmentation obtained through the k-fold verification of a single model. We evaluated the accuracy of the images outputted by the automatic contour segmentation by comparing them with the contour images obtained by manual contour delineation by radiation oncologists. Thus, we investigated the possibility of applying the automatic contour segmentation to clinical practice.

## MATERIALS AND METHODS

### Patients

The study design was approved by the Clinical Research Ethics Review Committee of our hospital (Approval No. 3081). Ninety-eight patients with stage IB to IVA cervical cancer (FIGO: International Federation of Gynecology and Obstetrics, 2008) who underwent axial-section DWI using MRI at our hospital between January 2005 and November 2013 were included. The patients provided written consent for the use of imaging data for research purpose.

### Outline of this study

**Fig. 1:**
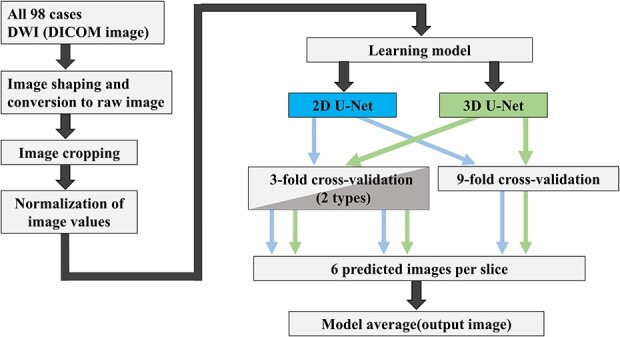
Outline of our study on automatic contour segmentation of cervical cancer sites based on diffusion-weighted MRI.

The study performed automatic contour segmentation of primary cervical cancer based on MRI DWI. First, we preprocessed the images of the 98 cases acquired in the Digital Imaging and Communications in Medicine (DICOM) format, including image formatting, raw data conversion, image cropping and normalization. Next, we created a learning model for the automatic contour segmentation. We used 2D U-Net and 3D U-Net as the learning models. Six learning models were created by combining the two U-Net models and three learning models with k-fold cross-validation, and six prediction images were acquired per case. The final output image is the model average of the six acquired images. Fig. 1 shows the outline of this study.

### Object images

The images of the object were axial sections of the DWI taken using the SIGNA HDx 3 T and SIGNA EXCITE 1.5 T (General Electric Medical Systems, Waukesha, WI, USA). The breakdown of the number of cases for each MR scanner was as follows: 67 cases for SIGNA HDx 3 T and 31 cases for SIGNA EXCITE 1.5 T. For the initial cases, SIGNA EXCITE 1.5 T was used; when the equipment was upgraded, SIGNA HDx 3 T was used. The imaging parameters were as follows: a slice thickness range of 5–8 mm, b value of 800 s/mm^2^, matrix size of 256 × 256 and pixel size range of (1.25 × 1.25) to (1.6406 × 1.6406) mm^2^. These images were extracted in the DICOM format for each case and outputted in raw format with the image size (number of images × height × width) set to 64 × 128 × 128 and 16-bit signed integer. In this study, a radiation oncologist, having 31 years of experience, contoured the GTV of cervical cancer and defined the tumor area using the Eclipse (Varian Medical Systems, Inc., Palo Alto, CA, USA) treatment planning system. During the contouring process, the GTV was delineated by checking the three sections of the MRI DWI and T2-weighted images for each case. The contoured image was created by binarizing the tumor area information from the DICOM structure format, i.e. the pixel values corresponding to the tumor and other areas were set to 1 and 0, respectively. The contour image was outputted as raw data in the same manner as the original DWI. This contour plotting image is the same size as the DWI, with good correspondence between geometric position and cross-section.

### Image preprocessing

**Fig. 2:**
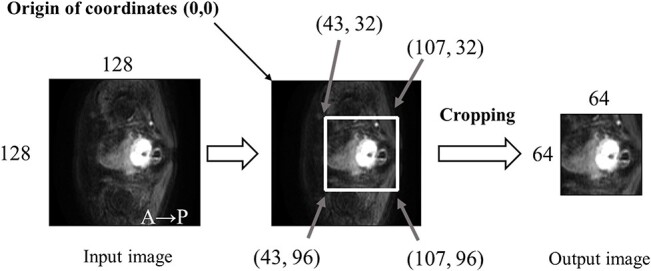
Flow of raw DWI data and contour information trimmed horizontally and vertically to reduce the computational cost.

The DWI and raw data, which serve as contour information, were cropped in terms of both the length and width to reduce the computational cost. Fig. 2 shows an overview of the cropping process. As shown, the transverse DWI is rotated 90° to the left, and the input and output images in the figure are the images taken before and after cropping, respectively. The DWI and contouring images per case were resized to 64 × 64 × 64 using this process. The resized images were used for the automatic contour segmentation. The conversion from the DICOM format and DICOM structure format to raw format was performed using MATLAB (2016b) (MathWorks, Natick, MA, USA).

The region of interest (ROI) for the cropping area was set by adopting a range that would include the tumor in all cases. By setting the ROI of the trimming region, the calculation cost for automatic contour segmentation can be reduced.

For the resized DWI, equation (1) was used to normalize the pixel values of each case. The normalization process was performed on 3D voxel data for each case. Here, pixel_in_ is the pixel value before processing, pixel_out_ is the pixel value of the image after processing, mean is the mean value of the pixel values in the image data and *σ* is the standard deviation:

(1) \begin{equation*} {\mathrm{Pixel}}_{\mathrm{out}}=\frac{{\mathrm{pixel}}_{\mathrm{in}}-\mathrm{mean}}{\sigma} \end{equation*}

### U-net structure

**Fig. 3:**
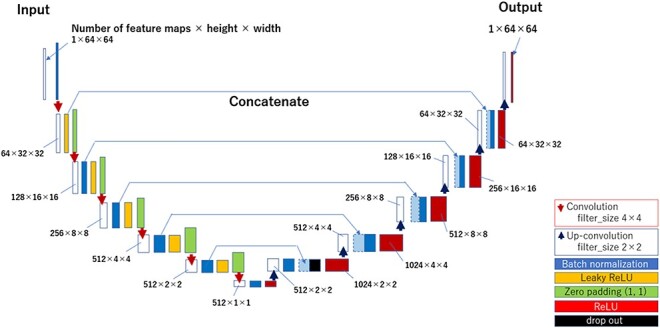
Structure of the 2D U-Net model.

The automatic contour segmentation in the 2D U-Net was performed on a per-image basis. We used one 2D image for the input images, each DWI was resized to 64 × 64 in height and width in ‘Object images.’ Fig. 3 shows the structure of the 2D U-Net used in this study. The 2D U-Net structure includes a convolutional layer [17], a reverse convolutional layer [17], a rectified linear unit (ReLU) layer [18], a leaky rectified linear unit (LeakyReLU) layer [19], a batch normalization layer [20] and a dropout layer [21]. The left half of the U-Net structure is called the reduced path, and the right half of the U-Net structure is called the extended path, where the image is reduced and extended, respectively.

We mainly performed operations using the batch-normalization layer, LeakyReLU layer, and convolutional layer of the reduced path. In the batch-normalization layer, the pixel values of the feature map were normalized for each batch. In the LeakyReLU layer, activations using LeakyReLU were performed on the pixel values of the feature map. The alpha value of the LeakyReLU was set to 0.2. The convolutional layer is computed with a filter size of 4 × 4, stride 2, and padding (1, 1) such that the size of the output is set to half the size of the input image in terms of the height and width. In the reduction pass, the convolution operation was repeated six times to reduce the output image size from 64 × 64 to 1 × 1.

The extended path uses a batch-normalization layer, an ReLU layer and a reverse convolutional layer. In the expansion pass, after the normalization process in the batch-normalization layer, we concatenated the feature maps of the same size from the reduction pass and then operated using ReLU. Thereafter, the output aspect size is expanded to twice the input aspect size by computing it in the reverse convolution layer with a filter size of 2 × 2 and a stride of 2. By repeating this inverse convolution operation six times, the image size was reduced to 1 × 1 in the reduction pass and was expanded to 64 × 64 pixels. A dropout layer was set in the expansion pass to suppress overtraining. The ratio in the dropout layer was set to 0.5. The final image with a size of 64 × 64 was used as the predicted image for the 2D U-Net.

In 3D U-Net, an automatic contour segmentation was performed on a voxel-by-voxel basis. The DWI were used as input for each case, with the image size set to 64 × 64 × 64 in object images—the 3D U-Net structure is used as a 3D extension of the 2D U-Net structure [15, 22]. The convolutional layer was computed with a filter size of 4 × 4 × 4, stride 2 and padding (1, 1, 1), so that the number of images and the height and width of the output were each set to half the input. The image size was reduced from 64 × 64 × 64 to 1 × 1 × 1 by repeating the convolution operation six times in the reduction pass.

In the expansion pass, the number of images and the height and width of the output were set to twice the input by computing with a filter size of 4 × 4 × 4 and a stride of 2 in the inverse convolution layer. In the expansion pass, the image size was increased from 1 × 1 × 1 to 64 × 64 × 64 by repeating the inverse convolution operation six times. The final image with a size of 64 × 64 × 64 was used as the predicted image for the 3D U-Net.

**Fig. 4:**
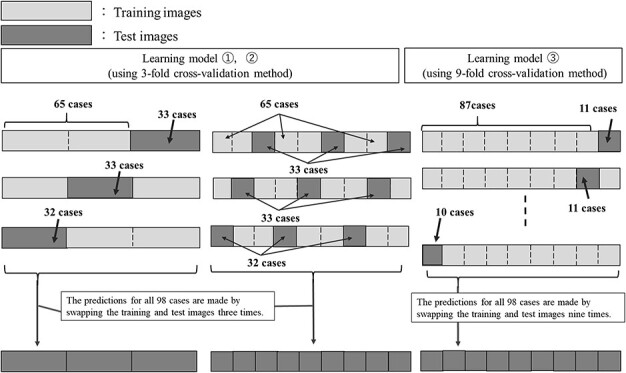
Outline of the selection process of training and test images for the three learning models (two types of 3-fold cross-validation methods and 9-fold cross-validation methods).

### Learning method

The DWI and GTV contour images used for the automatic contour segmentation were divided into training and testing images for subsequent prediction, respectively, for each case. For DWI, the images used for training were designated as training images and those used for testing were designated as test images. The GTV contour image was used as the correct answer image for the training and evaluation of the automatic contour segmentation. The correct answer images were divided into the correct answer images used for training, which corresponded to the training target images. The correct answer images for testing were used to evaluate the prediction accuracy after predicting the test images.

In the k-fold cross-validation, the used images are divided into k groups, of which k-1 are used for training, and the remaining one is used for training verification or prediction [23]. The k-fold cross-validation method has an advantage in the small cohort size because all the used images can be verified by swapping the training and verification images k times. In this study, we used a total of 98 cases. The k-fold cross-validation method was used for training models to predict all the 98 cases. Sixty-five cases were used as training images and 33 cases were used as test images in the two training models using the 9-fold cross-validation method. The two models with the 9-fold cross-validation method used 65 training images and 33 test images, whereas the two models with the 3-fold cross-validation method used 87 training images and 11 test images. The two models with the 3-fold cross-validation method differ in their selection of the training and test images. Fig. 4 shows an overview of the selection methods for the training and test images employed by the three training models.

**Fig. 5:**
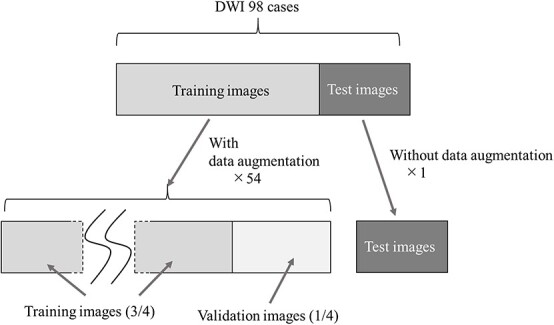
Overview of data expansion for training images and corresponding correct images. In this training model, the data were augmented 54 times by combining the translation of 2 pixels each in the vertical and horizontal directions, rotation of 5° in the image center axis in the clockwise–counterclockwise directions and left–right flipping.

During training, the training image and the corresponding correct image were augmented, and a quarter of them were used as images for training validation. In this training model, the data were augmented 54 times by combining the translation of 2 pixels each in the vertical and horizontal directions, rotation of 5° in the image center axis in the clockwise–counterclockwise directions and left–right flipping. Fig. 5 shows an overview of the data augmentation in the training image.

**Fig. 6:**
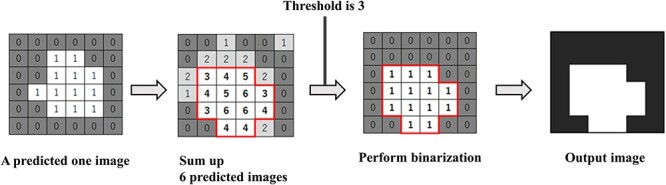
Flow to final image output by model averaging. The threshold value was set to 3 for this process. That is, if the summed values exceeded 3, the pixel was regarded as within gross tumor volume (GTV), and the total value is less than 3, the pixel was regarded as the outside of GTV.

After automatic contour learning, the image obtained by automatic contour segmentation using the test image was regarded as the predicted image. The batch size was set to 70 epochs and 64 batch sizes for the 3D U-Net, and five epochs and 64 batch sizes for the 2D U-Net. For all the training models, Adam [24] was used as the optimizer, and the learning rate was set to 0.001. The computer used for the automatic contour segmentation had a GeForce RTX 2080 graphics processing unit (GPU) (NVIDIA Corporation, Santa Clara, CA, USA). Python 3.6 was used as the programming language, and Keras 2.3.1 and TensorFlow-GTP 1.15 were used.

We used the Dice similarity coefficient (DSC):

(2) \begin{equation*} Dice=\frac{2\times \left|A\cap B\right|}{\left|A\right|+\left|B\right|} \end{equation*}

as the objective function, where Dice denotes the DSC, and *A* and *B* denote the predicted and correct image tumor regions, respectively [25]. The DCS has been used in many automatic contour segmentation studies [25]. For the 2D U-Net, the objective function was evaluated using the 2D image data of one image unit of the predicted image and the correct image. For the 3D U-Net, that was evaluated from the 3D image data of one case of the predicted image and the correct image. The DSC was also used as the index of the prediction accuracy for the evaluation of the test data.

### Model average

In this study, six predictive images per case were obtained by applying three different learning models to both 2D U-Net and 3D U-Net for the output images. Model averaging was performed on the six predicted images of the GTV contours obtained. Specifically, each pixel was summed and thresholder. The threshold value was set to 3 for this process. That is, if the summed values exceeded 3, the pixel was regarded as within GTV, and the total value is less than 3, the pixel was regarded as the outside of GTV. The image obtained by the model-averaging process was used as the final output image. Fig. 6 shows the process of outputting the final image by model averaging.

### Single model

The output of the final image was obtained by three k-fold validation models using 2D U-Net and 3D U-Net as a single model separated from the model average, which was obtained by the proposed multiple k-fold validation technique. Three k-fold validation models were used for 2D U-Net and 3D U-Net: two models with 3-fold cross-validation and one model with 9-fold cross-validation.

**Fig. 7:**
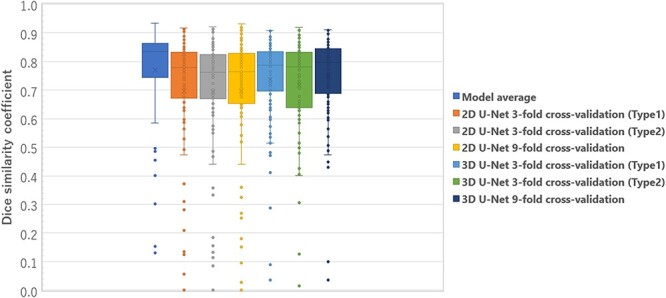
Box plots results of the DSC comparison between single model and model average with three k-fold validation models for 2D U-Net and 3D U-Net.

### Evaluation method

As described above, to evaluate the agreement between the predicted and correct images, we used the DSC. The DSC calculation for the final output image and the correct answer image were performed on the 3D image data per case.

Since an evaluation based on DSC alone would result in evaluation bias, we used Hausdorff distance (HD) to evaluate the result. Any point A on the correct image can reach any point B on the predicted image by advancing along with the least HD, and any point B on the solution image can reach any point A on the predicted image by advancing along with the least HD, which is expressed by equation 3. HD [mm] was calculated by multiplying the result obtained from the equation by the pixel size [mm/pixels] for each case.

(3) \begin{equation*} HD\left(\mathrm{A},\mathrm{B}\right)\left[\mathrm{pixels}\right]={\max}_{\mathrm{a}\in \mathrm{A}}\left({\min}_{\mathrm{b}\in \mathrm{B}}\left(\mathrm{d}\left(\mathrm{a},\mathrm{b}\right)\right)\right) \end{equation*}

### Comparison between single model and model average

To evaluate the usefulness of the automatic contour segmentation of cervical cancer by model averaging with the proposed multiple k-fold validation technique, we used the DSC results of automatic contour segmentation, which were obtained by three k-fold validation models based on 2D U-Net and 3D U-Net.

## RESULTS

The computational time required in the training was approximately 6 h for training using the 2D U-Net and approximately 15 h for training using the 3D U-Net. In the prediction step, it took approximately 5 min including the summation up the six predicted images to obtain the final output image after the binarization process.

### Comparison by single model and model average

Fig. 7 shows the box plots of DSC comparison between the single model with three k-fold validation models for 2D U-Net and 3D U-Net and model average. The 25th percentile, median and 75th percentile DSC of the model average are better than all single model results. In addition, there were fewer outliers in the model average.

### Model average results

**Fig. 8:**
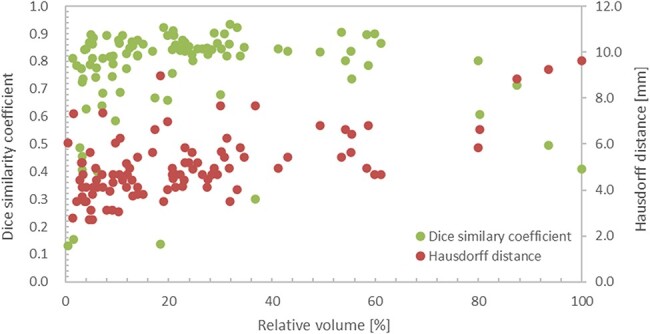
Results of the model averages of DSC and HD for the output and correct images for all 98 cases studied.

**Fig. 9:**
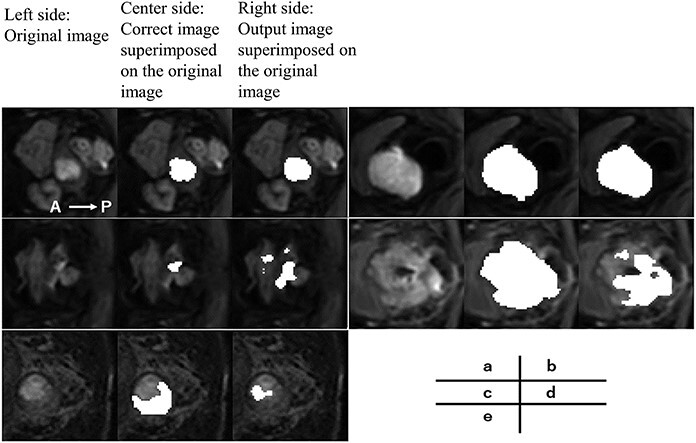
Example of the output image. On the left side is the original image. At the center is the correct image superimposed on the original image. On the right side is the output image superimposed on the original image. The DSC and HD are 0.88 and 4.1 mm for case (a), 0.91 and 5.4 mm for case (b), 0.13 and 6.1 mm for case (c), 0.41 and 9.6 mm for case (d) and 0.14 and 9.0 mm for case (e). In case (d) there is severe necrosis in the tumor, and in case (e) there is a uterine retained blood cell.

Fig. 8 shows the DSC and HD results of the model averages of the output image and the correct answer image for all the 98 cases studied. The first (left) vertical axis represents the DSC, whereas the second (right) axis represents the HD [mm]. Tumor volume was defined as the sum of the cross-sectional areas of the tumor at each cross-section, and relative volume [%] was defined as the normalized value of 100% for the case with the largest tumor volume (the maximum absolute volume of the tumor is 3500 mm2 with a relative volume of 100%). The DSC ranged from 0.13 to 0.93 (median 0.83, mean 0.77). Among these, 84 had DSC ≥ 0.65, and the remaining 14 had a DSC range of 0.13–0.64. Fourteen cases included those with a small maximum cross-sectional area of the tumor, retained uterine hematoma associated with the tumor and heterogeneous intracavitary concentration due to necrosis. A radiation oncologist made the diagnosis of a retained hematoma and necrosis inside the uterus. Cases with a small maximum cross-sectional area included those with a maximum diameter < 40 mm. Fig. 9 shows the output images obtained. The values of HD obtained from this study ranged from 2.7 to 9.6 mm, with a median of 4.7 mm and 90th percentile of 6.8 mm.

## DISCUSSION

This study incorporated k-fold cross-validation and model averaging into the learning model for automatic contour segmentation [26]. To the best of our knowledge, no study has applied automatic contour segmentation with model averaging to a GTV training model. The advantage of the k-fold cross-validation used in this study is that, as mentioned earlier, the study images can be replaced and automatic contour segmentation can be performed. Therefore, it could predict all the 98 acquired cases. Because more cases can be handled as the final output image, the overall trend of the learning model can be checked and evaluated. Therefore, it can be extended to a more general model. As shown in Fig. 7, the25th percentile, median and 75th percentile DSC and variability of the model averages increased from all the single model results. In addition, outliers and variability were reduced using the model adopted in this study, unlike when using the single model. Therefore, it is possible to achieve highly accurate automatic contour segmentation by using multiple k-fold validation, and the usefulness of the model developed in this study is demonstrated.

A disadvantage is that the number of training sessions for automatic contour segmentation increases, ultimately increasing the training time. Ensemble learning is a method that uses multiple learning models to produce an output while, bagging based on the random samplings combines the results of multiple learning models in parallel. From the results of multiple predictions, a region with a high degree of confidence can be outputted, and the effects of random noise can be removed. However, it is challenging to remove errors when the output images of many of the training models used for bagging contain the same errors. In this study, the colorectal region in the image shown in Fig. 9(c) was misrecognized as a tumor region by many of the learning models, resulting in a deviation from the correct image and a decrease in the DSC. In the future, in addition to U-Net, we plan to build a learning model that obtains predictive images from multiple networks, such as SegNet [27] and PSPNet [28] and perform merging and binarization.

The median DSC of the output images of all 98 cervical cancer cases was 0.83. In a study on automatic contour segmentation using U-Net, Wang *et al.* [12] used 100 CT images of 125 cervical cancer cases as training images and the remaining 25 as test images. They reported that the DSC of preventive CTV was approximately in the range of 0.8–0.9 (mean value: 0.86). Although it is necessary to consider the differences in the images used and the segmentation regions, one of the DSC characteristics of the output images in this study in comparison with previous studies is that there are several cases whose values were significantly lower than the average case. The cases with low DSCs tend to have small tumor cross-sectional areas, uterine hematoma and necrosis inside the tumor. For cases with a small tumor cross-sectional area, a slight deviation or noise can cause a significant decrease in the DSC based on the calculation method employed [25]. Based on the results of some of the output images from this study, there were several cases where normal tissue near the uterus and stools in the colon were misidentified as tumor areas. It was difficult to separate the hematoma in the tumor area because its pixel value was close to the tumor area. In the image shown in Fig. 9(e), a part of the retained hematoma was misidentified as a tumor, and the tumor area was not recognized; the DSC was 0.13, which is much lower than those in other cases. In the case of necrosis, the pixel values of the necrotic areas inside the tumor were not uniform, making it difficult to obtain accurate output in some cases. In particular, the hollow region inside the necrosis was depicted as a tumor in the correct image. However, it was not recognized as a tumor in the output image because of the low pixel values. Although there were some cases where the DSC was low due to certain factors, 50 out of the 98 outputs had a DSC accuracy ranging from 0.7 to 0.9.

The HDs reported by Wang et al. [12] ranged from 10 to 20 mm for CTVs of cervical cancer, including the prophylactic lymph node region, and approximately 3–10 mm for OARs of the bladder, femoral head, colon and other regions. Because prophylactic CTVs are, by definition, larger than GTVs and have different shapes, it is not easy to compare the evaluation of HD for automatic contour segmentation in the same way for both. Therefore, we do not compare the results of automatic contour segmentation of the GTVs in this study with the HD results of Wang *et al.* [12] for CTVs. The HD in this study showed that 90% of all cases fell within the range of 2.7–6.8 mm; however, there were nine cases with an HD value greater than 6.8 mm. The OARs of the bladder, femoral head, colon, reported by Wang *et al.*, were relatively similar in size to the GTV in this study; therefore, we compared them with the results of HD. The evaluation of HD in the automatic contour segmentation accuracy of GTV obtained in this study was comparable to the OAR of the bladder, femoral head and colon reported by Wang *et al* [12].

Four of these nine cases had DSCs in the range of 0.73 to 0.86, and the DSCs of these cases were highly consistent. These four cases were characterized by misidentification of normal tissues such as those of the uterus in the cross-section of the limbic area of the tumor and insufficient segmentation of the limbic area of the tumor, which resulted in low evaluation accuracy when considering HD. In contrast, near the center of the tumor, the segmentation accuracy was high and the evaluation accuracy when using DSC was high. Contrary to the four cases described above, we found six cases in which the DSC was below 0.65 and the HD match rate was higher than average. This suggests that in cases with a small relative volume of tumor, a small deviation in automatic contour segmentation caused a decrease in DSC, which is a measure of overall agreement; however, this decrease was not significant for HD, which evaluates the deviation in terms of distance. In the learning model used in the study, an averaging model was adopted to prioritize the removal of the effects caused by noise. However, we believe that when the relative volume of the tumor is small, the overlapping area in the averaging process becomes smaller, resulting in automatic contour segmentation with a smaller volume.

Therefore, although some corrections, such as noise, are necessary, this learning model can be useful in actual clinical practice as a tool to support radiation oncologists’ contouring work while significantly reducing their work time.

There are several limiting factors in this study. As the goal is to achieve highly accurate automatic contour extraction using AI, the number of contour extractors was limited to one to reduce the effect of inter-contour variation in the correct image. However, as the contour extraction performed by a single radiation oncologist may contain potential bias, future investigations will therefore implement contour extraction by multiple radiation oncologists.

In previous studies on tumor prediction using U-Net, approximately 100–300 cases were used as training images for automatic contour segmentation [2, 3, 12]. However, in this study, since cases were collected from a single institution, only a total of 98 cases, including training images and test images, were included. The numbers of cases including necrosis and retained uterine hematoma were only 27 and 16, respectively. The number of cases used for training was even lower when the training and test images were separated. To obtain more versatile and accurate automatic contour segmentation, it is necessary to collect and train many diverse cases, including these cases. Based on the above considerations and the actual number of cases collected for research in Japan, we believe that 200–300 cases should be used for an automatic contour segmentation of cervical cancer GTVs in the future. Multicenter studies can be considered to increase the number of cases. However, in the case of a multicenter study, doctors other than radiation oncologists may perform contouring work. In this case, individual differences in GTV contouring may occur depending on the person performing the work. In automatic contour segmentation, the individual differences in contour delineation reduce the training accuracy and affect the output [13].

In this study, we used DWI, which delineates tumor contours, to prevent the influence of individual differences in contour delineation in anticipation of future multicenter studies. In addition to the contour mentioned above, it is important to note that various parameters, such as MRI model, magnetic field strength and b-value, at the time of DWI may change the images and affect the learning prediction. It is necessary to set specific standards for imaging, such as those mentioned above.

This study evaluated the accuracy of automatic contour segmentation for cervical cancer tumors using 2D U-Net and 3D U-Net. The results suggest that the use of AI for GTV contouring in 3D-CRT planning has a high potential in assisting treatment planners.

## CONFLICT OF INTEREST

The authors declare no conflicts of interest regarding the publication of this paper.

## PRESENTATION AT A CONFERENCE

A part of this work is submitted to the 34^th^ annual meeting of Japan High-precision Beam Radiotherapy Group.

## CLINICAL TRIAL REGISTRATION NUMBER

The Institutional Review Board approved this retrospective study of our institution (Approval No. 3081).

## References

[1] Long J, Shelhamer E, Darrell T. Fully convolutional networks for semantic segmentation. In Proceedings of the IEEE Conference on Computer Vision and Pattern Recognition, IEEE Aerospace and Electronic Systems Society: New York and USA. 2015, pp. 3431–40.

[2] Hasan MSK, Linte CA. A modified U-Net convolutional network featuring a nearest-neighbor re-sampling-based elastic-transformation for brain tissue characterization and segmentation. In Proceedings IEEE West N Y Image Signal Process Workshop IEEE Aerospace and Electronic Systems Society: New York and USA. 2018. 10.1109/WNYIPW.2018.8576421 PMC6583803 31218299

[3] Baid U, Talbar S, Rane S et al. A novel approach for fully automatic Intra-tumor segmentation with 3D U-Net architecture for Gliomas. Front Comput Neurosci 2020;14:1–11. 32132913 10.3389/fncom.2020.00010 PMC7041417

[4] Park J, Yun J, Kim N et al. Fully automated lung lobe segmentation in volumetric chest CT with 3D U-net: validation with intra- and extra-datasets. J Digit Imaging 2020;33:221–30. 31152273 10.1007/s10278-019-00223-1 PMC7064651

[5] Wakamatsu Y, Kamiya N, Zhou X et al. Semantic segmentation of eight regions of upper and lower limb bones using 3D U-Net in whole-body CT Images. Jpn J Radiol Technol 2020;76:1125–32. 10.6009/jjrt.2020_JSRT_76.11.1125 33229842

[6] Miura H, Ozawa S, Doi Y et al. Automatic gas detection in prostate cancer patients during image-guided radiation therapy using a deep convolutional neural network. Phys Med 2019;64:24–8. 31515026 10.1016/j.ejmp.2019.06.009

[7] National Cancer Center Information Service. https://ganjoho.jp/public/cancer/cervix_uteri/treatment.html (22February 2021, date last accessed).

[8] Young AV, Wortham A, Wernick I et al. Atlas-based segmentation improves consistency and decreases time required for contouring postoperative endometrial cancer nodal volumes. Int J Radiat Oncol Biol Phys 2011;79:943–7. 21281897 10.1016/j.ijrobp.2010.04.063

[9] Ramadaan IS, Peick K, Hamilton DA et al. Validation of Varian's SmartAdapt® deformable image registration algorithm for clinical application. Radiat Oncol 2015;10:73. 25889772 10.1186/s13014-015-0372-1 PMC4465143

[10] Walker GV, Awan M, Tao R et al. Prospective randomized double-blind study of atlas-based organ-at-risk autosegmentation-assisted radiation planning in head and neck cancer. Radiother Oncol 2014;112:321–5. 25216572 10.1016/j.radonc.2014.08.028 PMC4252740

[11] Mutic S, Dempsey JF. The ViewRay system: magnetic resonance-guided and controlled radiotherapy. Semin Radiat Oncol 2014;24:196–9. 24931092 10.1016/j.semradonc.2014.02.008

[12] Wang Z, Chang Y, Peng Z et al. Evaluation of deep learning-based auto-segmentation algorithms for delineating clinical target volume and organs at risk involving data for 125 cervical cancer patients. J Appl Clin Med Phys 2020;21:272–9. 10.1002/acm2.13097 PMC7769393 33238060

[13] Chao KS, Bhide S, Chen H et al. Reduce in variation and improve efficiency of target volume delineation by a computer-assisted system using a deformable image registration approach. Int J Radiat Oncol Biol Phys 2007;68:1512–21. 17674982 10.1016/j.ijrobp.2007.04.037

[14] Pötter R, Tanderup K, Kirisits C et al. The EMBRACE II study: the outcome and prospect of two decades of evolution within the GEC-ESTRO GYN working group and the EMBRACE studies. Clin Transl Radiat Oncol 2018;9:48–60. 29594251 10.1016/j.ctro.2018.01.001 PMC5862686

[15] Ronneberger O, Fischer P, Brox T. U-Net: convolutional networks for biomedical image segmentation, MICCAI 2015. Part III, LNCS 2015;9351:234–41.

[16] Çiçek Ö, Abdulkadir A, Lienkamp SS et al. 3D U-Net: learning dense volumetric segmentation from sparse annotation. Proc. Medical Image Computing and Computer-Assisted Intervention–MICCAI 2016;424–32.

[17] Dumoulin V, Visin F. A guide to convolution arithmetic for deep learning arXivpreprint arXiv:1603.07285. 2016 January 12, 2018; preprint: not peer reviewed.

[18] Agarap AF. Deep learning using rectified linear units (ReLU) arXiv preprint arXiv:1803.08375. 2018 February 08, 2019; preprint: not peer reviewed.

[19] Xu B, Wang N, Chen T et al. Empirical evaluation of rectified activations in convolutional network arXiv preprint arXiv:1505.00853. 2015 November 30, 2015; preprint: not peer reviewed.

[20] Ioffe S, Szegedy C. Batch normalization: accelerating deep network training by reducing internal covariate shift arXiv:1502.03167. 2015 March 03, 2015; preprint: not peer reviewed.

[21] Srivastava N, Hinton G, Krizhevsky A et al. Dropout: a simple way to prevent neural networks from overfitting. JMLR 2014;15:1929–58.

[22] Ye R, Liu F, Zhang L. 3D depthwise convolution: reducing model parameters in 3D vision tasks arXiv:1808.01556. 2018 August 07, 2018; preprint: not peer reviewed.

[23] Blum A, Kalai A, Langford J. Beating the hold-out: bounds for k-fold and progressive crossvalidation. In Proceedings of the International Conference on Computational Learning Theory,International Conference on Computational Learning Theory: Nordkirchen, Germany 1999.

[24] Kingma D, Ba J. Adam: a method for stochastic optimization. In Proceedings of International Conference on Learning Representations, 3rd International Conference on Learning Representations: San Diego, CA, USA. 2015.

[25] Brock KK, Mutic S, McNutt TR et al. Use of image registration and fusion algorithms and techniques in radiotherapy: Report of the AAPM Radiation Therapy Committee Task Group No. 132. Med Phys 2017;44:e43–76. 28376237 10.1002/mp.12256

[26] Breiman L. Bagging predictors. Machine Learning 1996a;26:123–40.

[27] Badrinarayanan V, Kendall A, Cipolla R. Segnet: a deep convolutional encoder-decoder architecture for image segmentation arXiv:1511.00561. 2015 October 12, 2016; preprint: not peer reviewed. 10.1109/TPAMI.2016.2644615 28060704

[28] Zhao H, Shi J, Qi X et al. Pyramid scene parsing network. In Proceedings of the IEEE Conference on Computer Vision and Pattern Recognition, Conference on Computer Vision and Pattern Recognition (CVPR): Honolulu, HI, USA. 2017.

